# D_1_-A-D_2_ Conjugated Porous Polymers Provide Additional Electron Transfer Pathways for Efficient Photocatalytic Hydrogen Production

**DOI:** 10.3390/molecules30102190

**Published:** 2025-05-16

**Authors:** Zheng-Hui Xie, Yu-Jie Zhang, Jinhua Li, Shi-Yong Liu

**Affiliations:** 1 Jiangxi Provincial Key Laboratory of Functional Crystalline Materials Chemistry, School of Chemistry and Chemical Engineering, Jiangxi University of Science and Technology, Ganzhou 341000, China; stillonge@outlook.com (Z.-H.X.); 15079698527@163.com (Y.-J.Z.); 2 School of Chemical Engineering, Guangdong University of Petrochemical Technology, Maoming 525000, China

**Keywords:** π-conjugated porous polymers, direct C–H arylation polymerization, photocatalysis, D1-A-D2 conjugated polymer, visible-light-driven hydrogen evolution

## Abstract

The strategic design of donor–acceptor (D-A) conjugated porous polymers has emerged as a pivotal methodology for advancing efficient photocatalytic hydrogen evolution. However, conventional D-A polymeric architectures face inherent limitations: excessively strong acceptor units may lower the LUMO energy level, compromising proton (H^+^) reduction capability, while weak D-A interactions result in inadequate light-harvesting capacity and insufficient photogenerated electrons, ultimately diminishing photocatalytic activity. To address these challenges, we developed a new D1-A-D2 conjugated porous polymer (CPP) system. The strategic incorporation of a secondary donor benzothiophene (DBBTh) unit enabled precise bandgap engineering in D_1_-A-D_2_ CPPs. Experimental results demonstrate that DBBTh integration significantly enhances both light absorption efficiency and proton reduction ability. Under visible-light irradiation (λ > 420 nm), the Py-BKh1 photocatalyst achieved a hydrogen evolution rate (HER) of 10.2 mmol h^−1^ g^−1^ with an apparent quantum yield (AQY) of 9.5% at 500 nm. This work provides a groundbreaking paradigm for designing high-performance organic photocatalysts.

## 1. Introduction

Since Fujishima’s group [1] reported TiO_2_ for water splitting under ultraviolet (UV) light (λ > 290 nm) in 1972, an increasing number of researchers have focused on photocatalytic water splitting, leading to the discovery of numerous photocatalysts. However, most of these are inorganic materials, while organic photocatalysts remain rarely reported [2,3,4,5]. A paradigm shift emerged in 1985, Yanagida’s group [6] reported the use of poly(p-phenylene) (PPP) for UV-driven photocatalytic hydrogen evolution, marking the first demonstration of conjugated porous polymers as photocatalysts and revealing the potential of organic semiconductors in hydrogen production. Subsequently, in 2006, Wang’s group [7] reported graphitic carbon nitride (g-C_3_N_4_) for solar-driven hydrogen evolution, significantly advancing global research on organic semiconductor-based photocatalytic hydrogen generation. Finally, Shi’s group [8] reported the donor–acceptor (D-A) molecular design strategy—prevalent in organic solar cells—into photocatalysis, achieving groundbreaking progress. Building on decades of research, a wide range of D-A conjugated polymer photocatalysts has now been reported [9,10,11,12].

D-A conjugated polymers (CPs) are a class of macromolecular materials composed of π-conjugated chains formed by alternating donor (D) and acceptor (A) units. These CPs are assembled from organic monomers of non-metallic elements, and the structural diversity of available monomers enables the preparation of D-A CPs with tailored configurations. Notably, the D-A molecular architecture plays a decisive role in tuning their physicochemical properties [13,14]. This stems from the strong electron push–pull interaction between D and A units, where extended π-conjugation narrows the bandgap, enhancing light-harvesting capability and solar energy utilization [15]. Additionally, this electronic interaction promotes efficient exciton dissociation into free charge carriers, which migrate along predefined pathways to the acceptor. For instance, our group [16] reported enhanced photocatalytic activity through BTSO_2_-EDOT-based D-A CPs. The strong D-A effect amplified the optoelectronic response and accelerated exciton dissociation into mobile electrons, effectively suppressing electron–hole recombination. Under visible light, this system achieved a hydrogen evolution rate (HER) of 0.95 mmol h^−1^/6 mg with an apparent quantum yield (AQY) of 13.6% at 550 nm. The structural tunability of CPs allows surface modification via functional group incorporation. For instance, introducing alkoxy chains significantly improves hydrophilicity, enhancing interfacial reactions and photocatalytic efficiency [17]. For example, Zwijnenburg’s group [18] reported that grafting TEG (triethylene glycol) side chains onto CPs markedly increased hydrophilicity and swelling capacity, promoting water adsorption and accelerating surface reactions. This modification achieved a HER of 72.5 mmol h^−1^ g^−1^ under visible light. However, studies have indicated that the introduction of additional alkoxy chains may lead to the disruption of system planarity or reduction in chromophore density [19].

Despite the capability of D-A structured CPs to facilitate rapid exciton dissociation and enhance charge carrier separation, their molecular design presents significant challenges. Photocatalysts must satisfy the thermodynamic requirement where the lowest unoccupied molecular orbital (LUMO) energy level of the material lies more negative than the reduction potential of H_2_O/H_2_. Excessively strong acceptor units in D-A polymers may excessively lower the LUMO energy, rendering the CPs thermodynamically insufficient to drive hydrogen evolution. Conversely, weak D-A interactions compromise light-harvesting efficiency, limiting photogenerated electron density and diminishing photocatalytic activity. Consequently, the rational engineering of CPs with optimized bandgap widths and precisely aligned LUMO and highest occupied molecular orbital (HOMO) energy levels constitutes a critical step toward enhanced photocatalytic activity [20,21,22,23,24,25,26,27,28,29].

Introduction: A third component to regulate the monomer feeding ratio for achieving continuous and precise modulation of the bandgap has emerged as an important strategy to enhance the photocatalytic activity of D-A polymers [30,31,32,33,34,35,36,37,38,39,40]. For instance, Cooper’s group [41] reported a series of conjugated microporous polymers (CMPs) with tunable optical bandgaps (1.94–2.95 eV) by adjusting the feeding ratio of pyrene (Py) and benzene (Ph) monomers. Systematic investigation revealed the impact of monomer composition on the light absorption range and photocatalytic hydrogen production performance. Notably, CP-CMP10 (Py:Ph = 1:1) achieved a HER of 174 μmol h^−1^ g^−1^ under visible light (λ > 420 nm) irradiation without requiring metal co-catalysts. This work marked the first demonstration of continuous modulation of the optical bandgap in organic polymers, breaking the traditional limitations of fixed bandgaps in inorganic materials. Inspired by natural systems, Jin’s group [42] reported covalent triazine frameworks (CTFs) by incorporating carbazole as the donor unit, triazine as the primary acceptor (A_1_), and benzothiadiazole as a secondary acceptor (A_2_) to construct D-A_1_-A_2_ CTFs. Under visible light irradiation, the optimized polymer exhibited an AQY of 22.8% at 420 nm and a HER of 19.3 mmol h^−1^ g^−1^, with Pt as co-catalyst. This design highlights the synergistic advantages of multi-component systems in advancing photocatalytic efficiency.

Inspired by the aforementioned studies, we herein strategically constructed a series of ternary conjugated porous polymers (CPPs) with D-A configuration or D_1_-A-D_2_ configurations—denoted as Py-BKh0, Py-BKh1, Py-BKh2, and Py-BKh3—through atom economical C–H/C–Br cross-coupling reactions. These polymers were synthesized, using 1,3,6,8-tetrabromopyrene (TBPy) as the primary donor (D_1_), bis(2-thienyl)ketone (BTK) as the acceptor (A), and 5,5′-dibromo-2,2′-bithiophene (DBBTh) as the secondary donor (D_2_), by precisely modulating the feeding ratio between the primary and secondary donors. Experimental results demonstrate that the incorporation of the secondary donor significantly enhances the light responsiveness and reducing capacity of the CPPs. The secondary donor DBBTh facilitates the formation of additional electron transport pathways, promoting photogenerated charge separation and thereby improving photocatalytic activity. Under visible light irradiation (λ ≥ 420 nm), the optimized CPPs achieved a HER of 10.2 mmol h^−1^ g^−1^ and an AQY of 9.5% at 500 nm. Our work provides novel insights into the molecular-level design of advanced D-A-type organic photocatalysts, offering a fresh perspective for developing high-efficiency photocatalytic systems.

## 2. Results and Discussion

### 2.1. Synthesis and Structural Characterization

**Figure 1:**
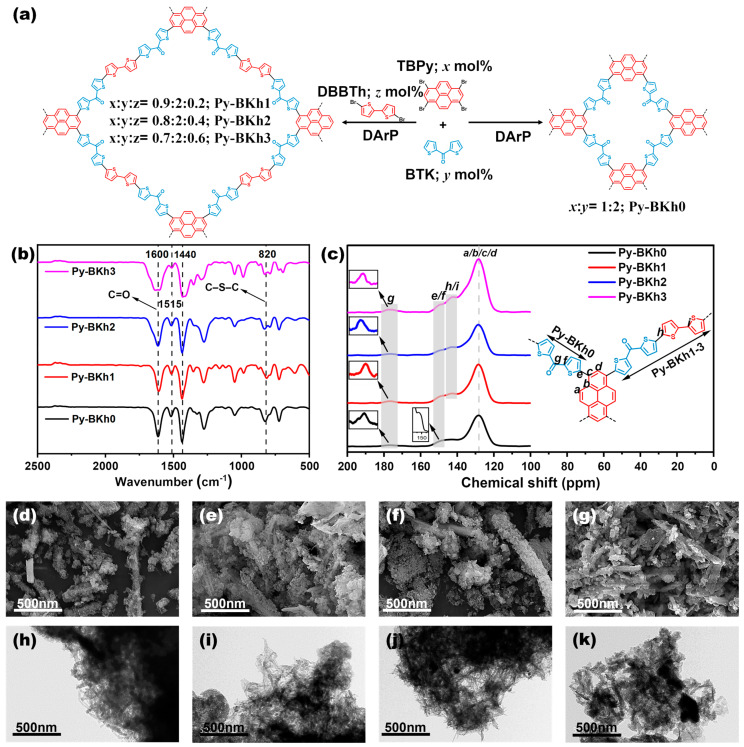
(**a**) Synthetic routes of Py-BKh0, Py-BKh1, Py-BKh2, and Py-BKh3; (**b**) FTIR spectra and (**c**) solid-state ^13^C NMR spectra of all polymers; SEM images of Py-BKh0 (**d**), Py-BKh1 (**e**), Py-BKh2 (**f**), and Py-BKh3 (**g**); and TEM images of Py-BKh0 (**h**), Py-BKh1 (**i**), Py-BKh2 (**j**), and (**k**) Py-BKh3.

We synthesized the D-A-type binary conjugated polymer Py-BKh0 via a green, atom-economical C–H/C–Br cross-coupling reaction [16,35,36,37,43,44,45], utilizing 1,3,6,8-tetrabromopyrene (TBPy) with a rigid planar framework and bis(2-thienyl)ketone (BTK) featuring a strong electron-withdrawing carbonyl group. Subsequently, 5,5′-dibromo-2,2′-bithiophene (DBBTh) was introduced as a secondary donor (D_2_) to construct the ternary conjugated porous polymers (CPPs) with a D_1_-A-D_2_ configuration. Here, BTK serves as the electron acceptor (A), while TBPy and DBBTh act as electron donors (D). By strategically adjusting the relative content of the secondary donor (DBBTh) and primary donor (TBPy), three conjugated porous polymer samples—Py-BKh1, Py-BKh2, and Py-BKh3—were synthesized. The specific reaction pathway is illustrated in Figure 1a. The chemical structures of the synthesized polymers were characterized using Fourier-transform infrared (FT-IR) spectroscopy. As shown in Figure 1b and Figure S1, the polymers share similar spectral features due to their identical building blocks. A broad vibration peak at 1600 cm^−1^ is attributed to the C=O stretching vibration, while absorption signals at 1400 cm^−1^ and 1515 cm^−1^ correspond to aromatic ring skeletal stretching vibrations. The broad peak at 820 cm^−1^ arises from the C–S–C characteristic vibration in the thiophene groups. Figure 1c and Figure S2 show the solid-state ^13^C NMR spectra of the polymers, with carbon atoms labeled as “*a*-*i*”. All polymers exhibit a signal at ~177 ppm corresponding to the carbonyl group (carbon g) in BTK, while the signal at ~128 ppm belongs to the carbon atoms (a, b, c and d) in the BTPy unit. The tertiary carbon signals at the α-position of thiophene in BTK appear at ~150 ppm (carbons e and f), and the signal at ~142 ppm is attributed to the tertiary carbons at the α-position of DBBTh (carbons h and i). Notably, the DBBTh-associated signals are absent in Py-BKh0 but emerge in Py-BKh1, Py-BKh2, and Py-BKh3 with progressively increasing intensity, confirming the successful incorporation of DBBTh. The above analysis demonstrates that the trends in FTIR and ^13^C NMR spectra are consistent, indicating the successful synthesis of target CPPs with varied DBBTh content.

To investigate the effect of DBBTh content on the polymers’ micromorphology, scanning electron microscopy (SEM) and transmission electron microscopy (TEM) were employed. As shown in Figure 1c–j, SEM images reveal that Py-BKh0 exhibits a mixed structure of micron-scale rods and particles with irregular dispersion, whereas Py-BKh1, Py-BKh2, and Py-BKh3 display denser layered structures. This indicates that the introduction of DBBTh modifies the surface morphology of Py-BKh0, and the layered structure facilitates the exposure of more active sites. TEM images show irregular aggregates of similar size across all samples. Figure 1g–i exhibit fibrous aggregation, while Figure 1j shows a bulk-like aggregation, suggesting that low DBBTh content has a minimal impact on the aggregation state of Py-BKh0, but higher levels induce morphological changes. These results demonstrate that the adjustment of a secondary donor (DBBTh) content effectively modulates the micromorphology of the polymers.

### 2.2. Optical and Electrochemical Properties

**Figure 2:**
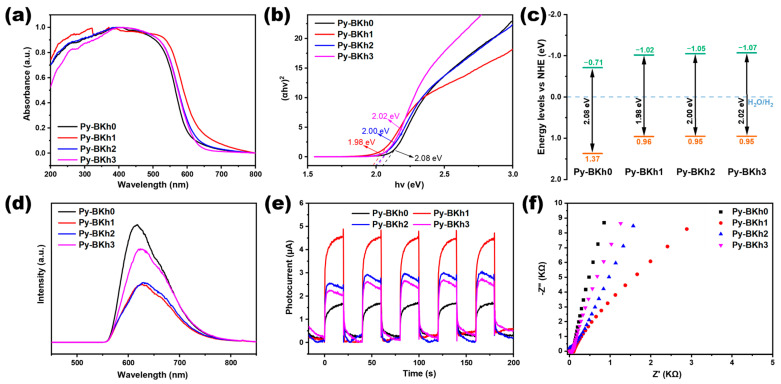
(**a**) UV–vis DRS. (**b**) Tauc-plot maps. (**c**) Energy band alignments. (**d**) Steady-state PL spectra. (**e**) TPR under visible light irradiation. (**f**) EIS of all CPPs.

To investigate the structure–property relationships of the polymers, we first performed ultraviolet–visible diffuse reflectance spectroscopy (UV-Vis DRS) on the four conjugated polymeric samples (Py-BKh0, Py-BKh1, Py-BKh2, and Py-BKh3). As shown in Figure 2a, all polymers exhibit strong absorption in the 300–600 nm range, with absorption edges approaching 700 nm, which facilitates visible light harvesting for photocatalytic reactions. Notably, the ternary Py-BKh1, Py-BKh2, and Py-BKh3 exhibit more redshift phenomena than that of binary Py-BKh0, with Py-BKh1 demonstrating the most bathochromic shift. This indicates that the introduction of the secondary donor DBBTh into Py-BKh0 positively enhances light absorption, and the D_1_-A-D_2_ ternary polymer configuration improves photon utilization. Although increasing the DBBTh content beyond optimal levels reduces light absorption, the precise control of DBBTh incorporation achieves broad absorption spectra. The optical bandgaps of the polymers, determined via the Kubelka–Munk equation using (αhv)² vs. hv plots (Figure 2b), are calculated as 2.08, 1.98, 2.00, and 2.02 eV for Py-BKh0, Py-BKh1, Py-BKh2, and Py-BKh3, respectively. The observed bandgap narrowing followed by widening aligns with the absorption profiles, confirming that adjustment of the TBPy: DBBTh ratio enables tuning light absorption ranges and bandgaps. Cyclic voltammetry (CV) was employed to determine the lowest unoccupied molecular orbital (LUMO) energy levels (Figure S3). After conversion to the standard hydrogen electrode, the LUMO levels are measured at −0.71, −1.02, −1.05, and −1.07 eV for Py-BKh0, Py-BKh1, Py-BKh2, and Py-BKh3, respectively. Using the relationship E_HOMO_ = E_LUMO_ + Eg, the highest occupied molecular orbital (HOMO) levels are calculated as 1.37, 0.96, 0.95, and 0.95 eV, respectively. The derived band structures are illustrated in Figure 2c. All polymers exhibit LUMO levels thermodynamically capable of driving hydrogen evolution. Notably, the LUMO levels of Py-BKh1, Py-BKh2, and Py-BKh3 are elevated compared to Py-BKh0, indicating enhanced driving forces for hydrogen production in the ternary polymers. Combining UV–Vis DRS, Kubelka–Munk analysis, and CV data, we demonstrate that the introduction of the secondary donor DBBTh narrows the optical bandgap to enhance light absorption, simultaneously elevating LUMO levels to strengthen photocatalytic reduction capability. These synergistic effects would have a significant impact on improving hydrogen evolution performance, highlighting the potential of molecular engineering for optimizing polymer-based photocatalysts.

The separation and transfer of photogenerated electron–hole pairs (e^−^-h^+^) are key processes in photocatalysis. To further investigate the charge carrier separation efficiency of the polymers, we characterized the photophysical properties of the four conjugated organic polymer samples using steady-state photoluminescence (PL) spectroscopy, transient photocurrent response (TPR), and electrochemical impedance spectroscopy (EIS), as shown in Figure 2d–f. Steady-state PL spectroscopy is widely used to evaluate the radiative recombination of photogenerated charge carriers [46,47,48,49]. A higher PL intensity typically indicates stronger radiative recombination and lower charge separation efficiency, which is detrimental to photocatalytic activity. Figure 2d displays the PL intensities with a sequence of Py-BKh0 > Py-BKh3 > Py-BKh2 > Py-BKh1 under 400 nm excitation. The strong PL signal of Py-BKh0 suggests severe electron–hole recombination and poor charge separation efficiency, limiting its photocatalytic activity. In contrast, the introduction of the secondary donor DBBTh significantly suppresses PL intensity, with Py-BKh1 showing the weakest emission. This confirms that the D_1_-A-D_2_ ternary polymeric architecture effectively reduces radiative recombination and enhances charge separation, thereby improving photocatalytic efficiency. TPR and EIS measurements were further conducted to evaluate charge separation dynamics. TPR analysis (Figure 2e) reveals that the ternary polymers (Py-BKh1–3) showed more enhanced photocurrent responses than that of Py-BKh0 under full-spectrum xenon lamp irradiation, indicating superior charge separation and transport capabilities in the DBBTh-modified polymers. EIS Nyquist plots (Figure 2f) demonstrate reduced charge transfer resistance for the ternary polymers, as evidenced by smaller semicircle radii. Py-BKh1 shows the smallest radius, corresponding to the lowest interfacial resistance and most efficient suppression of electron–hole recombination. These results align with the TPR results, confirming that the secondary donor DBBTh facilitates charge separation. Collectively, these characterizations demonstrate that the D_1_-A-D_2_ configuration promotes directional electron transfer toward acceptor units, enabling the efficient reduction of H^+^ to H_2_. This structural design synergistically enhances light absorption, charge separation, and catalytic activity, providing a robust strategy for developing high-performance polymeric photocatalysts.

### 2.3. Photocatalytic Hydrogen Production Performance

**Figure 3:**
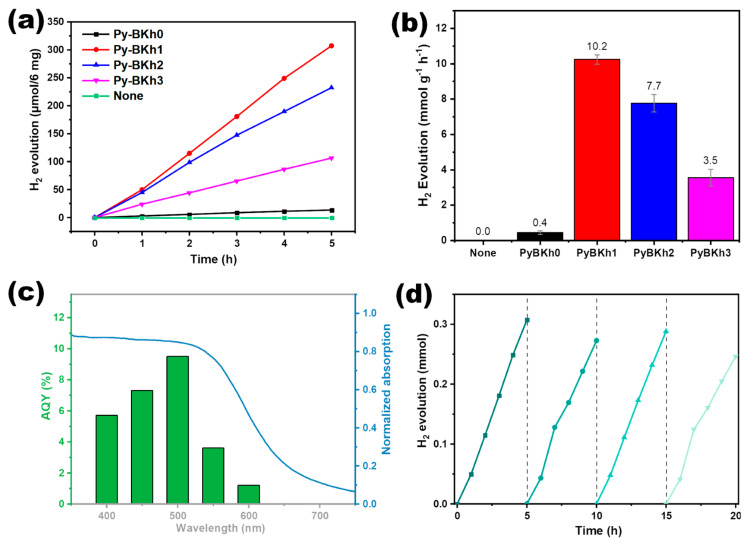
(**a**) Hydrogen production rate over time, (**b**) normalized HER for all CPPs, (**c**) AQY of Py-BKh1 at different wavelengths, (**d**) cyclic hydrogen production test for Py-BKh1.

To examine the structure–performance correlations of the polymers, photocatalytic hydrogen evolution tests were conducted on Py-BKh0, Py-BKh1, Py-BKh2, and Py-BKh3 without co-catalysts. Under the condition of visible light (λ ≥ 420 nm) irradiation, 6 mg polymeric catalysts, 1 M ascorbic acid as a sacrificial agent, and an NMP/H_2_O (*v*:*v* = 1:5) mixed solvent, the hydrogen evolution rates (HERs) of Py-BKh0, Py-BKh1, Py-BKh2, and Py-BKh3 were 2.7, 61.5, 46.5, and 21.3 μmol h^−1^, respectively (Figure 3a–b), corresponding to normalized HERs of 0.4, 10.2, 7.7, and 3.5 mmol h^−1^ g^−1^, and no hydrogen production was observed in the absence of photocatalyst and light. Although the HER initially increased and then decreased with higher DBBTh content, all ternary systems (Py-BKh1–3) outperformed the binary counterpart (Py-BKh0), achieving 25-, 19-, and 8-fold enhancements, respectively. This demonstrates that the secondary donor DBBTh enhances light harvesting and creates energy-level gradients to suppress charge recombination, thereby promoting charge separation and migration. Py-BKh1 exhibited the highest HER of 10.2 mmol h^−1^ g^−1^, highlighting the critical role of optimizing the TBPy:DBBTh ratio in tuning energy-level structures for improved photocatalytic activity.

To evaluate solar-to-hydrogen conversion efficiency, the apparent quantum yield (AQY) of Py-BKh1 was measured under monochromatic light (Figure 3c). Using a 6 mg catalyst with only water and ascorbic acid, AQYs reached 5.7%, 7.3%, 9.5%, 3.6%, and 1.2% at 400, 450, 500, 550, and 600 nm, respectively, consistent with its UV–vis absorption profile. The maximum AQY of 9.5% at 500 nm confirms efficient light utilization in the visible range and validates the photo-driven nature of hydrogen generation. Cycling tests assessed the stability of Py-BKh1 over 20 h (four cycles). As shown in Figure 3d, the HER gradually declined but retained ~80% of its initial activity, demonstrating robust stability. The slight activity recovery in the third cycle is attributed to the replenishment of the sacrificial agent (ascorbic acid), confirming that the catalyst retains structural integrity during prolonged operation. These results underscore the practical potential of the D_1_-A-D_2_ ternary design for sustainable photocatalytic hydrogen production. To check the residual Pd contents of the CPPs and its effect on HER, we altered the Pd catalyst to synthesize three batches of Py-BKh1 by using 2 mol%, 4 mol%, and 6 mol% Pd_2_(dba)_3_, which are accordingly denoted as Py-BKh1-2, Py-BKh1-4, and Py-BKh1-6, respectively. The ICP-MS test reveals that all CPPs exhibited almost equal residual Pt loading (~0.6 wt%, Table 1), which could be attributed to the saturation of Pd loaded on the CPPs after the identical post-treatment procedures. The hydrogen production tests on these three CPPs showed a slight increase in HER with an increase in Pd pre-catalyst loadings. This may be attributed to the increase in polymerization degrees due to the increased Pd loadings of the Pd catalyst. The effect of residual Pd and the polymerization degree on the photocatalytic performance explored here also coincides with previous studies [50].

**Figure 4:**
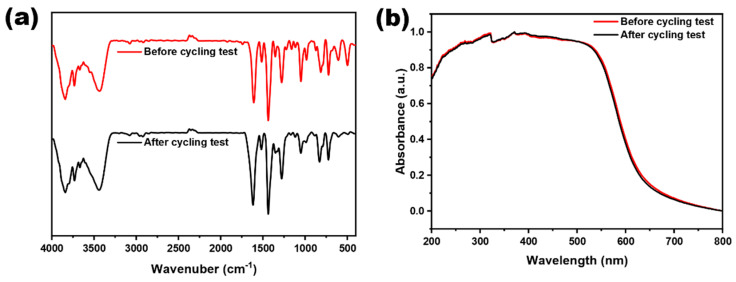
(**a**) UV−vis DRS and (**b**) FT-IR spectra of Py-BKh1 before and after recycling tests.

Meanwhile, the Py-BKh1 was recovered for structural analysis, and no observable changes occurred in the UV−vis and FT-IR spectra compared to the as-prepared one (Figure 4), manifesting the long-term stability of Py-BKh1 in the photocatalytic hydrogenation reaction. To enable comprehensive comparison, Table 2 summarizes the HER of Py-BKh1 and reference polymer photocatalysts. The results demonstrate that Py-BKh1 stands as one of the top-performing candidates among reported polymeric photocatalysts to date.

## 3. Materials and Methods

### 3.1. Methods

All of the starting materials and reagents were purchased from commercial suppliers (Aladdin, Shanghai, China) and used directly without further purification. Anhydrous toluene was pretreated with calcium hydride (CaH_2_) and freshly distilled.

FT-IR spectra were collected on a FT-IR spectrometer (Bruker ALPHA), and the KBr was mixed with the sample for sample preparation. Solid-state magic angle spinning ^13^C NMR measurements were carried out on a Bruker NEO WB 400M model 400 MHz NMR spectrometer at a MAS rate of 10 kHz. SEM images and TEM images of the polymers were obtained by scanning electron microscopy (SEM, MLA650F, Hillsboro, OR, USA) and transmission electron microscopy (TEM, FEI Tecnai G2 F20, USA). We recorded polymer photoluminescence (PL) conductivity by employing a HORIBA FL-1000 fluorescence spectrometer for solid powders. The photocatalytic output of hydrogen was quantitatively and qualitatively detected by gas chromatography (GC9790). The solid UV–visible absorption spectra of the synthesized polymers were detected by a UV-2600 spectrophotometer using BaSO_4_ as a substrate reference. Transient photoresponse tests and electrochemical impedance spectroscopy were performed using a three-electrode configuration electrochemical workstation (CHI650E/700E, Shanghai Huachen Co., Ltd., Shanghai, China), where transient photocurrents and electrochemical impedances were measured using a platinum electrode as the auxiliary electrode and a silver/silver chloride electrode containing saturated potassium chloride solution as the reference electrode. The polymer was ultrasonically dispersed with ethanol to form a suspension, which was then dripped onto ITO conductive glass to form a sample with an effective area of 0.6 cm × 0.6 cm, and tested in a 0.1 M aqueous sodium sulfate solution as the electrolyte. Cyclic voltammetry testing is performed by using an electrochemical workstation (CHI650E/700E, Huachen Co LTD, Shanghai, China). The Pd metal residue was obtained by Agilent ICP-MS 7700, weighed a fixed measure of the sample, with an added 3 mL of HCl and 1 mL of HNO_3_, microwave-heated at 180 °C for 30 min, and then transferred to a fixed volume for measurement. Elemental analyses of the polymers were obtained by Elimonta UNICUBE.

### 3.2. Synthesis of bis(2-thiophene)ketone, Py-BKh0, Py-BKh1, Py-BKh2, and Py-BKh3

Bis(2-thiophene)ketone: Firstly, the vacuum and argon substitution operation was repeated three times on the three-necked round-bottomed flask through the double-row tube, and, under the protection of argon atmosphere, AlCl_3_ (36.6 mmol, 4.9 g) and anhydrous CH_2_Cl_2_ (1.5 mL) were added sequentially, the reaction temperature was controlled in the range of 0–4 °C, and the content of the flask was stirred for 10 min. Then, 2-thiophenecarbonyl chloride (9.1 mmol, 2.0 g) was dissolved in anhydrous CH_2_Cl_2_ and added into a dropping funnel, slowly dripped into the reaction flask and stirred for 10 min. Finally, thiophene (19 mmol, 1.6 g) was dissolved in anhydrous CH_2_Cl_2_ and added to the dropping funnel, slowly dripped into the reaction flask and stirred for 20 min, returned to room temperature, and reacted overnight. The product was poured into a mixture of 2 M HCl and ice water, the organic phase was collected by extraction and dried with anhydrous Na_2_SO_4_, the solvent was removed by rotary evaporation under vacuum, and the product was purified by column chromatography to give a light-yellow solid in 80% yield (1.4 g). ^1^H NMR (400 MHz, Chloroform-d) δ 7.95 −7.87 (m, 2H), 7.74 −7.66 (m, 2H), and 7.22 −7.15 (m, 2H). ^1^H NMR spectra are shown in the Supporting Information.

Procedure A: A proportion of 1,3,6,8-tetrabromopyrene (TBPy), bis(2-thiophene)ketone (BTK), 5,5′-dibromo-2,2′-bithiophene (DBBTh), anhydrous cesium carbonate (Cs_2_CO_3_), valerianic acid (PivOH), tris(dibenzylidene acetone)dipalladium (Pd_2_(dba)_3_), and (*o*-methoxyphenyl)phosphine (P(*o*-MeOPh)_3_) were added into the Schlenk tube. The system was subjected to three cycles of vacuum and argon purging to ensure an oxygen-free environment. Anhydrous toluene was then added as the solvent under argon atmosphere, followed by three freeze–pump–thaw cycles to further remove residual oxygen. The reaction mixture was stirred continuously in an oil bath at 110 °C for 48 h. After the reaction, the crude product was cooled to room temperature and washed with dichloromethane, methanol, water and methanol, respectively, to remove small-molecule products and inorganic salts, and then dried under vacuum at 80 °C for 12 h to obtain the final product.

Py-BKh0: 1,3,6,8-tetrabromopyrene (1 eq, 133.3 mg), bis(2-thienyl)ketone (2 eq, 100.0 mg), Cs_2_CO_3_ (4 eq, 335.4 mg), PivOH (0.6 eq, 15.8 mg), catalyst Pd_2_(dba)_3_ (0.04 eq, 7.1 mg), and ligand P(*o*-MeOPh)_3_ (0.08 eq, 5.4 mg), reaction solvent anhydrous toluene of 6 mL. Following the description in Procedure A, the final product Py-BKh0 (137.0 mg, 90.7%) was obtained. IR (cm^−1^): 1600, 1515, 1400, and 820. ^13^C NMR (400 MHz, ppm) 177, 150, and 128.

Py-BKh1: 1,3,6,8-tetrabromopyrene (0.9 eq, 180.0 mg), bis(2-thienyl)ketone (2 eq, 100.0 mg), 5,5′-Dibromo-2,2′-bithiophene (0.2 eq, 25.0 mg), Cs_2_CO_3_ (4 eq, 335.4 mg), PivOH (0.6 eq, 15.8 mg), catalyst Pd_2_(dba)_3_ (0.04 eq, 7.1 mg), and ligand P(o-MeOPh)_3_ (0.08 eq, 5.4 mg), reaction solvent anhydrous toluene of 6 mL. Following the description in Procedure A, the final product Py-BKh1 (208.1 mg, 91.3%) was obtained. IR (cm^−1^): 1600, 1515, 1400, and 820. ^13^C NMR (400 MHz, ppm) 177, 150, 142, and 128.

Py-BKh2: 1,3,6,8-tetrabromopyrene (0.8 eq, 160.0 mg), bis(2-thienyl)ketone (2 eq, 100.0 mg), 5,5′-Dibromo-2,2′-bithiophene (0.4 eq, 50.0 mg), Cs_2_CO_3_ (4 eq, 335.4 mg), PivOH (0.6 eq, 15.8 mg), catalyst Pd_2_(dba)_3_ (0.04 eq, 7.1 mg), and ligand P(o-MeOPh)_3_ (0.08 eq, 5.4 mg), reaction solvent anhydrous toluene of 6 mL. Following the description in Procedure A, the final product Py-BKh2 (197.1 mg) was obtained. IR (cm^−1^): 1600, 1515, 1400, and 820. ^13^C NMR (400 MHz, ppm) 177, 150, 142, and 128.

Py-BKh3: 1,3,6,8-tetrabromopyrene (0.7 eq, 140.0 mg), bis(2-thienyl)ketone (2 eq, 100.0 mg), 5,5′-Dibromo-2,2′-bithiophene (0.6 eq, 75.0 mg), Cs_2_CO_3_ (4 eq, 335.4 mg), PivOH (0.6 eq, 15.8 mg), catalyst Pd_2_(dba)_3_ (0.04 eq, 7.1 mg), and ligand P(o-MeOPh)_3_ (0.08 eq, 5.4 mg), reaction solvent anhydrous toluene of 6 mL. Following the description in Procedure A, the final product Py-BKh3 (213.1 mg, 91.5%) was obtained. IR (cm^−1^): 1600, 1515, 1400, and 820. ^13^C NMR (400 MHz, ppm) 177, 150, 142, and 128.

## 4. Conclusions

Herein, we synthesized a donor (D)-acceptor (A) binary CPP via C–H/C–Br cross-coupling reactions using 1,3,6,8-tetrabromopyrene (TBPy) and bis(2-thienyl)ketone (BTK). Subsequently, a ternary D_1_-A-D_2_ CPP was constructed by incorporating 5,5′-dibromo-2,2′-bithiophene (DBBTh) as a secondary donor (D_2_). Under visible-light irradiation (λ > 420 nm), the Py-BKh1(D_1_:A:D_2_ = 0.9:2:0.2) photocatalyst exhibited a hydrogen evolution rate (HER) of 10.2 mmol h^−1^ g^−1^ with an apparent quantum yield (AQY) of 9.5% at 500 nm. Systematic characterization revealed that the introduction of DBBTh significantly enhanced the CPPs’ light-harvesting capability and proton (H^+^) reduction capacity, enabling stronger charge carrier generation. This study employs copolymerization and diversified linkage strategies to modify polymeric structures, emphasizing the critical roles of visible-light absorption and charge transport in photocatalytic hydrogen production. Our findings provide a rational design framework for optimizing polymer architectures and electronic properties, paving the way for enhanced photocatalytic activity.

## Figures and Tables

**Table 1:** Remaining Pd and HER of Py-BKh1 synthesized by different amounts of Pd catalysts.

Material	Pd_2_(dba)_3_^a^ (mol%)	W (Pd) ^b^ (wt%)	H_2_ Evolution Rate (mmol h^−1^ g^−1^)
Py-BKh1-2	2.0	0.6636	8.8
Py-BKh1-4	4.0	0.6154	10.2
Py-BKh1-6	6.0	0.6347	12.5

^a^ SBED-BT_0.5%_ synthesized using Pd_2_(dba)_3_ with different feed ratios; ^b^ The Remaining Pd in the polymers obtained by Agilent ICP-MS 7700.

**Table 2:** Summary of photocatalytic hydrogenolysis reactions of photocatalysts.

Pub Date	Materials	H_2_ Yield (mmol h^−1^ g^−1^)	AQY	Ref.
This Work	Py-BKh1	10.2	9.5% (500 nm)	-
2025.4	M-TiO_2_-7	1.3	-	[51]
2025.3	ST@BTTA-120	3.69	-	[52]
2025.3	PFBT-Pdots	2.23	-	[53]
2025.2	SCN-0.5	1.67	5.8% (420 nm)	[54]
2024.9	PyBT-COF-COOH	8.15	5.1% (420 nm)	[55]
2024.7	Py-T-BTDO-3	8.66	4.42% (420 nm)	[56]
2024.6	PDTPDPA-BP	2.16	-	[57]
2023.11	NPs-TpPa-1	1.13	13.5% (450 nm)	[58]
2023.9	Zn-Por-TPT	11.45	-	[59]

## Data Availability

Data are contained within this article and Supplementary Materials.

## Supplementary Materials

The following supporting information can be downloaded at https://www.mdpi.com/article/10.3390/molecules30102190/s1, Figure S1. Fourier exchanged infrared (FTIR) spectrograms of all polymers; Figure S2. Solid-state ^13^C NMR spectra of all polymers; Figure S3. CV curves of all polymers; Figure S4. Synthesis of bis(2-thienyl)ketone (BTK); Figure S5. ¹H NMR spectrum of BTK dissolved in CDCl_3_.
