# Supplementary material for: D1-A-D2 Conjugated Porous Polymers Provide Additional Electron Transfer Pathways for Efficient Photocatalytic Hydrogen Production
Source: Molecules. 2025 May 16;30(10):2190. doi: 10.3390/molecules30102190 (PMC12114361; doi:10.3390/molecules30102190)
Supplement: Supplementary file 1 [file molecules-30-02190-s001.zip › molecules-3593317-supplementary.pdf]

Supporting Information

D_1-A-D_2 Conjugated Porous Polymers Provide Additional Electron Transfer Pathways for Efficient Photocatalytic Hydrogen Production

Zheng-Hui Xie[1, †], Yu-Jie Zhang[1, †], Jinhua Li [1, *], and Shi-Yong Liu[1, 2 *]

[1] Jiangxi Provincial Key Laboratory of Functional Molecular Materials Chemistry, School of Chemistry and Chemical Engineering, Jiangxi University of Science and Technology, Ganzhou 341000, China; 6720210821@mail.jxust.edu.cn (H. Gong); xing_yu_qin2@sina.com (Y.-Q. Xing)

* Correspondence: lijh@jxust.edu.cn (J. Li); chelsy@jxust.edu.cn or chelsy@zju.edu.cn (S.-Y. Liu)

[2,] School of Chemical Engineering, Guangdong University of Petrochemical Technology, Maoming, Guangdong 525000, China.

† These authors contributed equally to this work

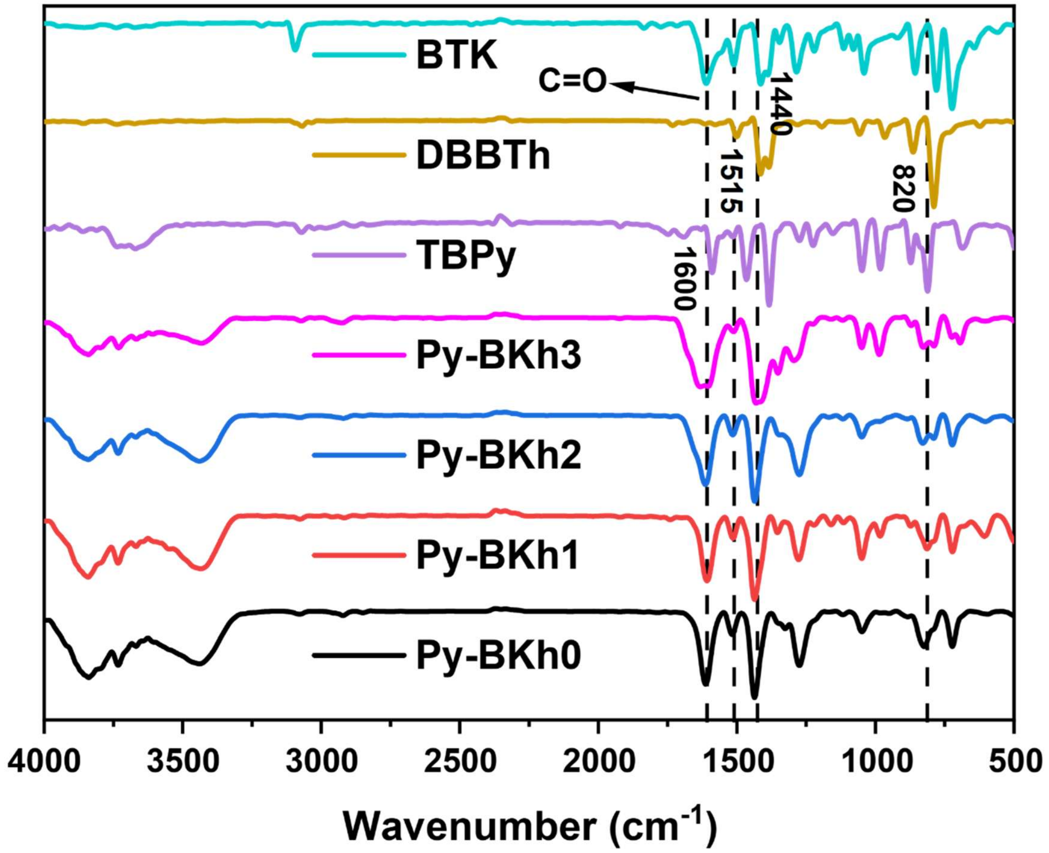

Figure S1. Fourier exchanged infrared (FTIR) spectrograms of all polymers.

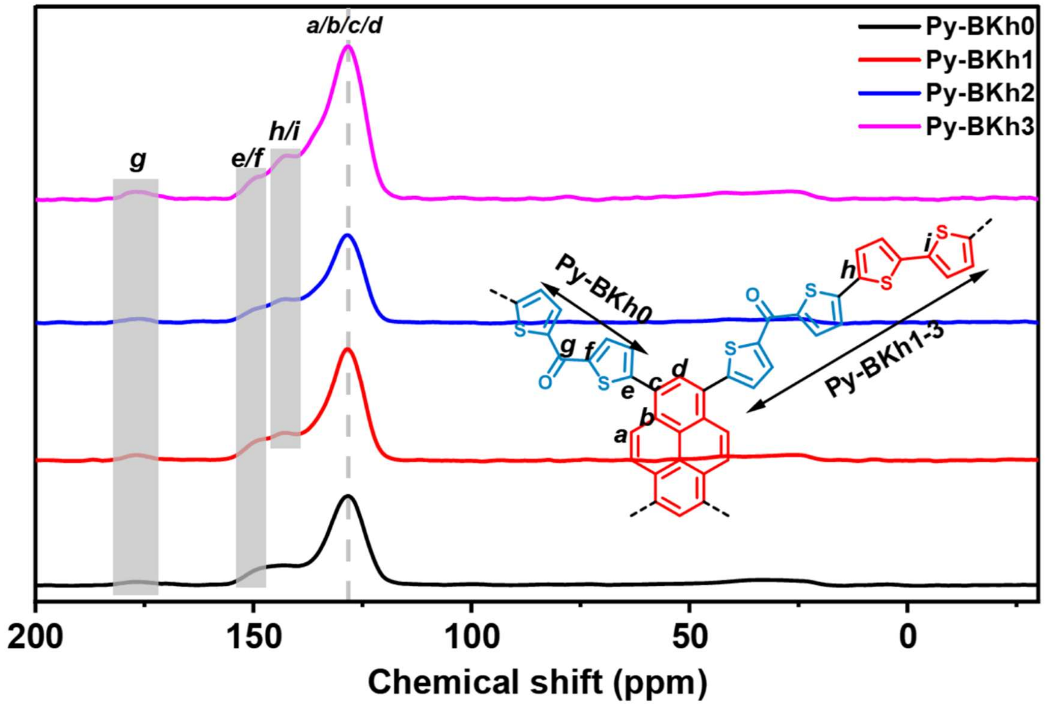

Figure S2. Solid-state ^{13}C NMR spectra of all polymers.

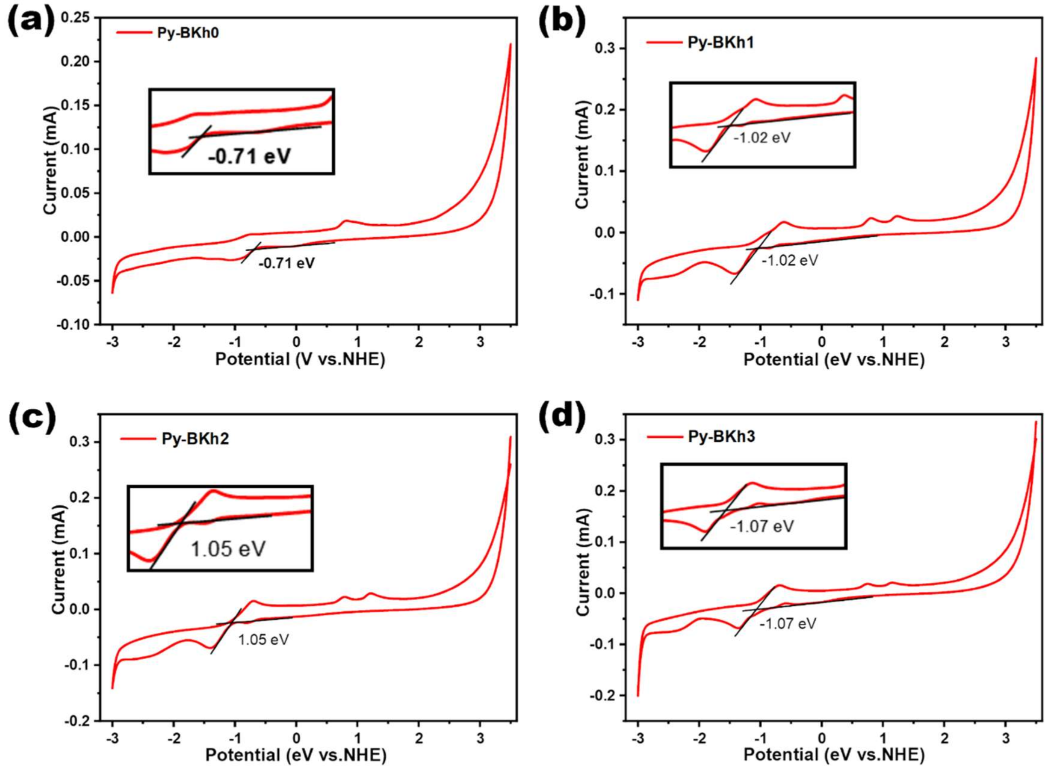

Figure S3. CV curves of all polymers.

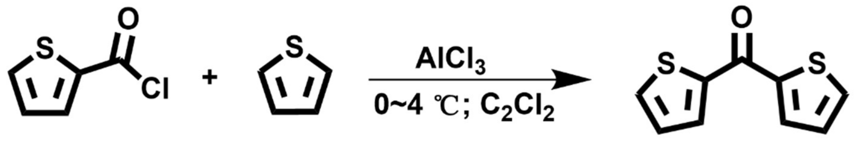

Figure S4. Synthesis of bis(2-thienyl)ketone (BTK).

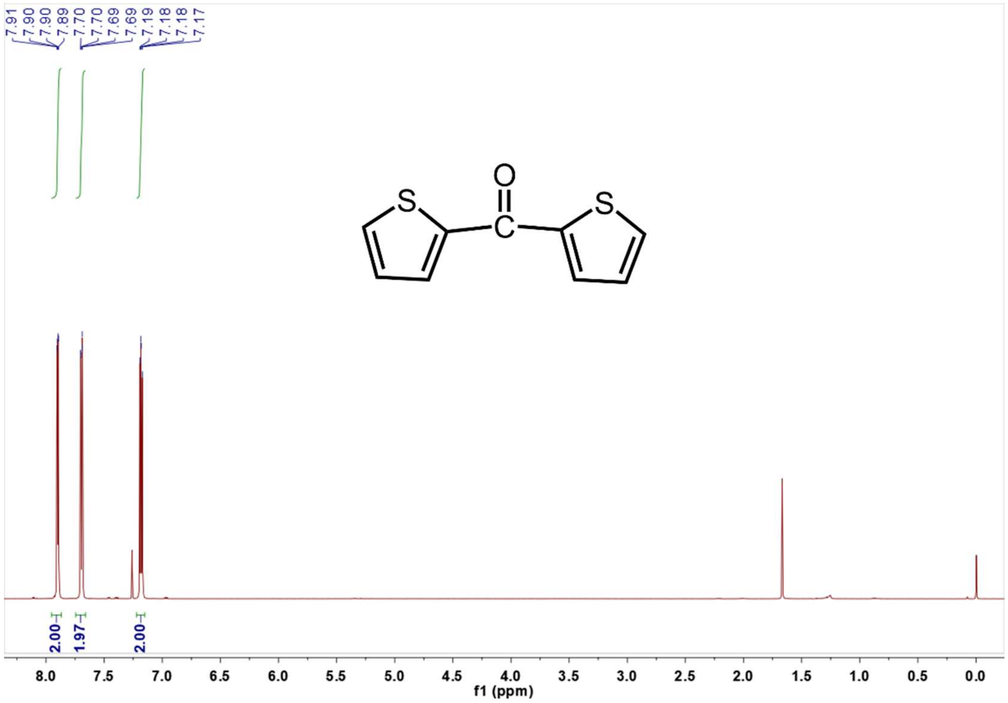

Figure S5. ^{1}H NMR spectrum of BTK dissolved in $CDCl_3$.
